# Supplementary material for: Altered microRNA Transcriptome in Cultured Human Liver Cells upon Infection with Ebola Virus
Source: Int J Mol Sci. 2021 Apr 6;22(7):3792. doi: 10.3390/ijms22073792 (PMC8038836; doi:10.3390/ijms22073792)
Supplement: Supplementary file 1 [file ijms-22-03792-s001.zip › Supplementary_File/D_ KEGG_Analysis_Results/16-30nt_pathway_Makona-96h-Huh7_vs_Control-96h-Huh7_down.mature_mirna_targets/hsa_EnrichmentScoreDotPlot.pdf]

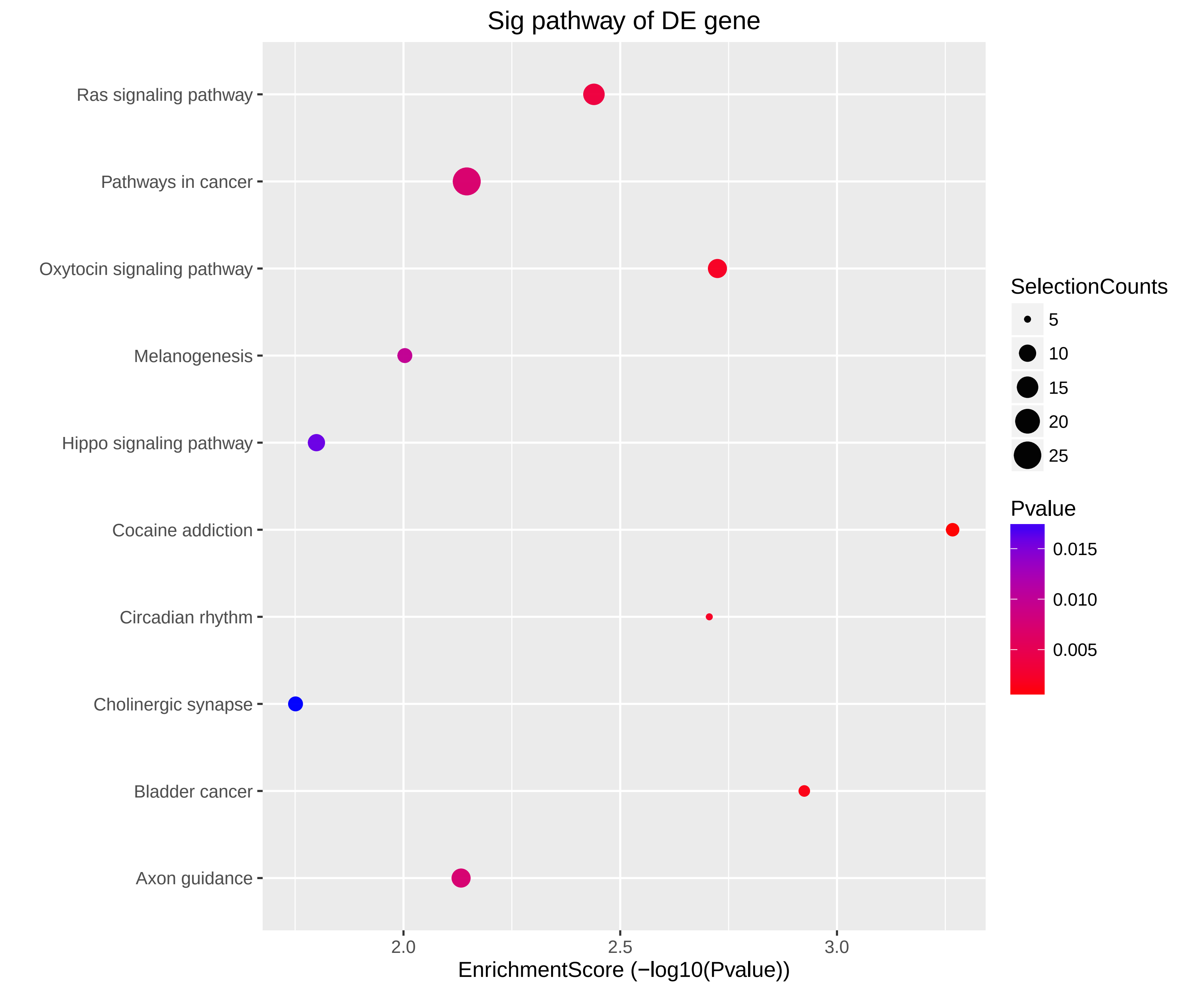
Sig pathway of DE gene
Ras signaling pathway
Pathways in cancer
Oxytocin signaling pathway
Melanogenesis
Hippo signaling pathway
Cocaine addiction
Circadian rhythm
Cholinergic synapse
Bladder cancer
Axon guidance
2.0
2.5
3.0
EnrichmentScore (−log10(Pvalue))
SelectionCounts
5
10
15
20
25
Pvalue
0.015
0.010
0.005
